# Revolutionizing migraine management: advances and challenges in CGRP-targeted therapies and their clinical implications

**DOI:** 10.3389/fneur.2024.1402569

**Published:** 2024-06-13

**Authors:** A. Özge, B. Baykan, Ş. Bıçakçı, M. Ertaş, A. Ç. Atalar, S. Gümrü, N. Karlı

**Affiliations:** ^1^Department of Neurology, Algology and Clinical Neurophysiology, Mersin University School of Medicine, Mersin, Türkiye; ^2^Department of Neurology and Clinical Neurophysiology, Istanbul Faculty of Medicine, Istanbul University, Istanbul, Türkiye; ^3^Department of Neurology, Faculty of Medicine, Cukurova University, Adana, Türkiye; ^4^Department of Neurology, University Health Sciences, Istanbul Physical Medicine and Rehabilitation Training and Research Hospital, Istanbul, Türkiye; ^5^Pfizer Pharmaceuticals, Istanbul, Türkiye; ^6^Department of Neurology, Faculty of Medicine, Uludag University, Bursa, Türkiye

**Keywords:** CGRP-targeted therapies, migraine management, clinical implications, gepants, monoclonal antibodies, personalized treatment

## Abstract

Migraine, a prevalent neurological disorder, affects approximately 14.1% of the global population and disproportionately impacts females. This debilitating condition significantly compromises quality of life, productivity, and incurs high healthcare costs, presenting a challenge not only to individuals but to societal structures as a whole. Despite advances in our understanding of migraine pathophysiology, treatment options remain limited, necessitating ongoing research into effective therapies. This review delves into the complexity of migraine management, examining the roles of genetic predisposition, environmental influences, personalized treatment approaches, comorbidities, efficacy and safety of existing acute and preventive treatments. It further explores the continuum between migraine and tension-type headaches and discusses the intricacies of treating various migraine subtypes, including those with and without aura. We emphasize the recent paradigm shift toward trigeminovascular activation and the release of vasoactive substances, such as calcitonin gene-related peptide (CGRP), which offer novel therapeutic targets. We assess groundbreaking clinical trials, pharmacokinetic and pharmacodynamic perspectives, safety, tolerability, and the real-world application of CGRP monoclonal antibodies and gepants. In the face of persisting treatment barriers such as misdiagnosis, medication overuse headaches, and limited access to specialist care, we discuss innovative CGRP-targeted strategies, the high cost and scarcity of long-term efficacy data, and suggest comprehensive solutions tailored to Turkiye and developing countries. The review offers strategic recommendations including the formulation of primary care guidelines, establishment of specialized outpatient clinics, updating physicians on novel treatments, enhancing global accessibility to advanced therapies, and fostering patient education. Emphasizing the importance of lifestyle modifications and holistic approaches, the review underscores the potential of mass media and patient groups in disseminating critical health information and shaping the future of migraine management.

## 1 Introduction

Migraine is a prevalent neurological disorder, affecting approximately 14.1% of the global population, with a higher incidence among females than males (1, 2). It is a debilitating condition that significantly impacts the patient’s quality of life, daily activities, productivity, and is associated with substantial healthcare costs (3, 4). The burden of migraine is not limited to the individual, but it also has a significant impact on their families and society as a whole, social psychological and economical (5). Despite substantial progress in understanding the pathophysiology of migraine, effective treatment options are still limited.

The complexity and heterogeneity of migraine’s etiology, pathophysiology, and treatment management pose significant challenges to developing effective therapies (6). Several factors, such as the interaction of genetic and environmental factors, hormonal changes, oxidative stress, inflammation, and neuronal hyperexcitability, have been implicated in migraine etiology and pathophysiology (7–9). It has been estimated that about 42% of migraines are inherited and relatives of cases with elevated pain scores, frequent attacks, early onset, migraine with aura faced a heightened risk (9). It is also hypothesized that tension type headache and migraine are two ends of the same spectrum in which transition in between can be seen (10–12) There are different subtypes of migraine, like migraine with and without aura, each with distinct clinical features and pathophysiology, which makes it challenging to treat (13). The drug treatment includes acute and preventive therapies, which aim to reduce the frequency, severity, and duration and burden of migraine attacks and improve the patient’s quality of life (14).

The understanding of the mechanisms underlying migraine has advanced in recent years and many potential targets for acute and preventive treatment have been identified (15, 16). The most recent hypothesis is the trigeminovascular activation resulting from the nociceptive signals originating from the meningeal vessels via trigeminal sensory branches which are then transmitted to cortical parts of the central nervous system. During this process several vasoactive substances including calcitonin gene-related peptide (CGRP) are released resulting in vasodilatation of the vessels and pain production. New therapeutic agents including CGRP monoclonal antibodies, anti-CGRP receptor antibodies, small-molecule CGRP receptor antagonists, gepants, ditans, and neuromodulation devices have shown promise in clinical trials (17–19). The development of these new treatments represent a significant unmet need in the field (20).

In this review article we aimed to;

- Explain the rationale of CGRP as the target of new migraine therapies.
- Review the major clinical trials, pharmacokinetic/dynamic insights, safety and tolerability profiles with real-world data (if available) of the CGRP monoclonal antibodies and gepants.
- Assess the clinical implications of new treatments targeting CGRP, opportunities and challenges for CGRP monoclonal antibodies and gepants launching.
- Provide specific recommendations for future treatment landscape in the context of Turkiye and developing countries.

## 2 Methods

An Expert Group Advisory Committee Meeting, involving 6 experts in neurology therapeutic area from Turkiye was convened in November 2022 to identify the scope of the literature search and to evaluate the resources. Each expert was either a member of an academic association, contributed to development of guidelines on the subject or published articles on migraine management. Prior to that meeting, a literature review was performed to highlight the evaluation of calcitonin gene-related peptide targeted therapies, current guidelines, obstacles to management and recommendations for effective migraine treatment from a global and regional Turkish perspective. To identify relevant articles, we searched the MEDLINE® (via the PubMed interface), Web of Science, Google Scholar and EMBASE databases. An electronic search of the literature published from 2000 to 2024 was conducted in these databases by using MeSH (Medical Subject Heading, Medline) and EMBASE terms, as well as free text words. The search included the terms “calcitonin gene-related peptide targeted therapies,” “current headache guidelines” “migraine management” and “migraine and Turkiye.” The inclusion criteria were: (1) peer-reviewed articles and scientific reports, (2) original articles, review articles and conference papers, including information about migraine management (3) publication between 2000 and 2024. The exclusion criteria were: (1) articles not published in either English or Turkish, and (2) case reports. The reference lists of all manuscripts were manually reviewed for additional eligible articles. Most recent and up to date publications were chosen. Relying upon this literature review, experts created a national perspective and developed brief recommendations for a future treatment landscape in Turkiye focused on migraine management.

## 3 Rational and evaluation of CGRP targeted therapies

**Figure 1:**
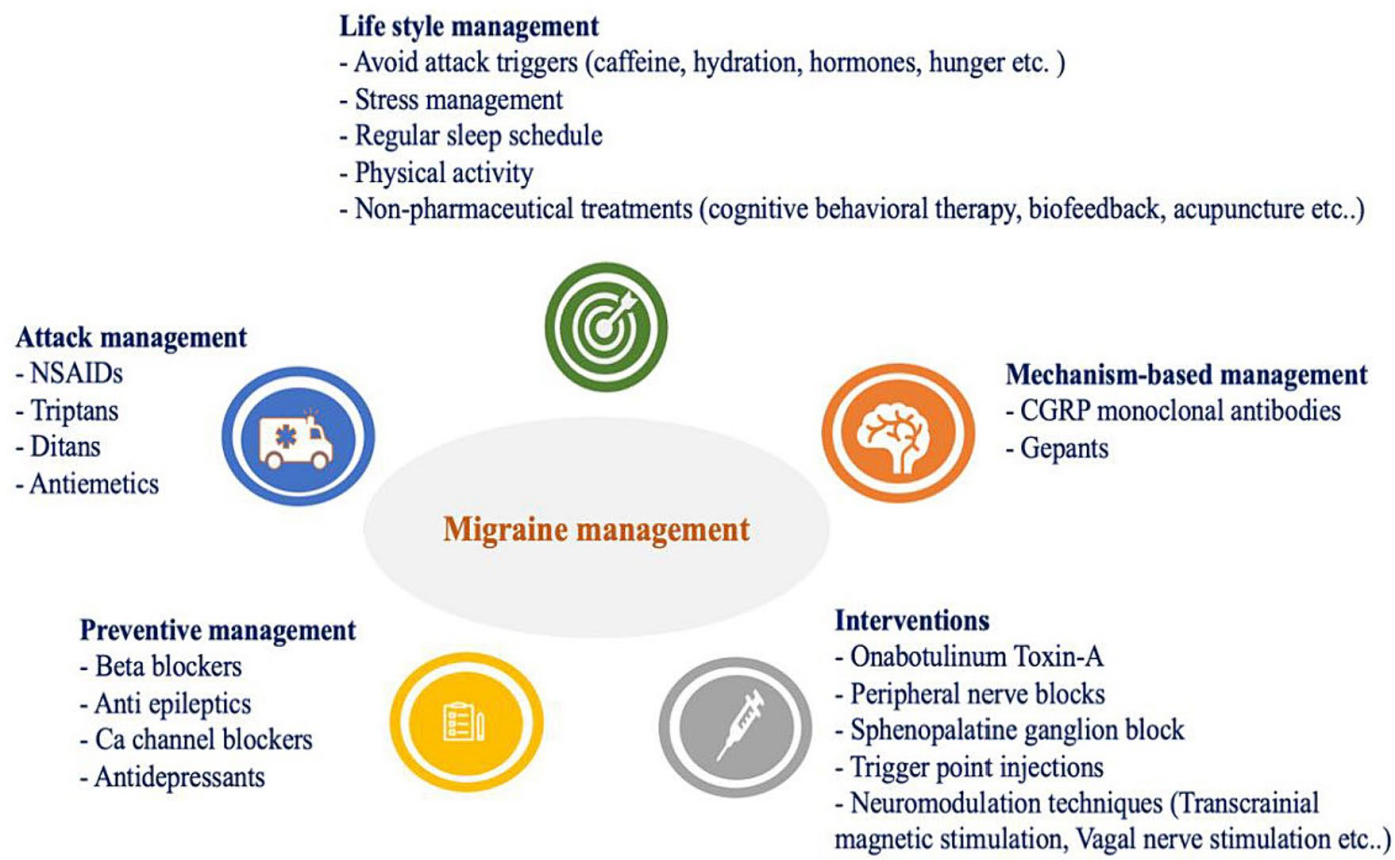
Current migraine management.

Standard conventional migraine treatment can be divided in to acute and preventive strategies both still inholding significant unmet needs. These include suboptimal optimization, limited efficacy, excessive reliance on acute treatment agents leading to medication overuse headache (MOH) and the absence of suitable therapeutic options for all individuals (21). This brings the need for more effective, tolerable therapies with fewer contraindications. Recent clinical trials have demonstrated the effectiveness of monoclonal antibodies targeting CGRP or its receptor in reducing the frequency and severity of migraines (19). Other promising approaches include neuromodulation techniques, such as transcranial magnetic stimulation and vagus nerve stimulation, which have shown efficacy in reducing migraine frequency and severity (22, 23). In addition, non-pharmacological treatments, such as cognitive behavioral therapy and mindfulness-based interventions, have also shown promise in reducing migraine frequency and improving quality of life (24, 25). This article mainly concentrates on CGRP targets, as they dominate the mechanism of action of recent acute and preventive treatment options. Current migraine management approaches are summarized in Figure 1.

**Figure 2:**
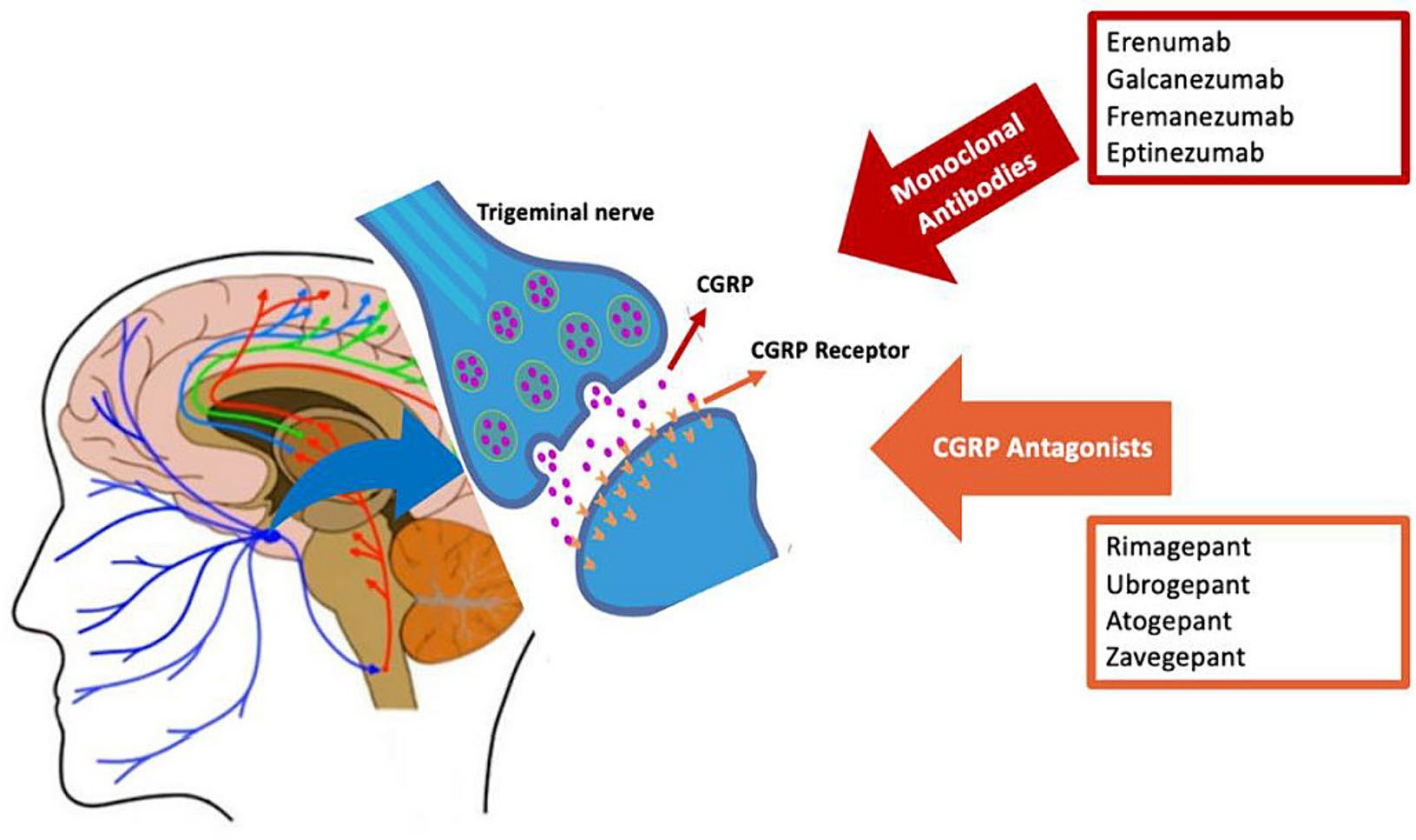
Calcitonin gene-related peptide (CGRP)-targeting drugs for migraine.

There are two main types of CGRP-targeted drugs (Figure 2): CGRP monoclonal antibodies, which are large-molecule CGRP receptor or ligand antagonists and gepants, which are small-molecule CGRP receptor antagonists (26).

### 3.1 Monoclonal antibodies

Monoclonal antibodies are highly selective for CGRP and CGRP receptors, leading to fewer side effects and drug interactions (27). Currently, there are four monoclonal antibodies available for the preventive treatment of migraine: erenumab, eptinezumab, fremanezumab, and galcanezumab.

Erenumab, an IgG2 CGRP receptor blocker, is the first monoclonal antibody approved for the preventive treatment of migraine in adults (28, 29). In two Phase II trials erenumab was found to be effective and tolerable in chronic migraine patients along with the extension studies (30–33). Phase III STRIVE, ARISE and LIBERTY studies showed similar efficacy and adverse effects on episodic migraine (34–37). Erenumab also showed favorable results in efficacy and tolerability against topiramate in a phase IV comparative study (38). Adverse effects include constipation, injection site reactions, muscle spasms, and pruritus also shown in real world studies (RWS) (35, 39–44). Ongoing research on erenumab is investigating the efficacy and safety of the drug in pediatric patients with episodic and chronic migraine (NCT03836040, NCT03832998). Additionally, a Phase IV trial (NCT04592952) is investigating the efficacy of erenumab in high-frequency episodic headaches. Several RWS have investigated the safety and effectiveness of erenumab, with nearly all studies showing similar results of a reduction in monthly migraine days (MMD) with increased quality of life (40, 42, 45–48). A large RWS study on the impact of erenumab on absenteeism, healthcare resource use, and comorbidities has been completed, but the results have not yet been published (NCT05375097).

Fremanezumab is the second monoclonal antibody approved, available in monthly doses of 225 mg or quarterly doses of 675 mg (28, 49). Phase II and phase III HALO studies demonstrated successful results for both episodic and chronic migraine (50–53). The FOCUS trial also demonstrated favorable results (54). Recruiting studies of fremanezumab on sleep improvement (NCT04693533) and two other studies for the preventive treatment of episodic and chronic migraine in pediatric patients are still ongoing (NCT04530110, NCT04464707). RWS of fremanezumab have demonstrated its efficacy and tolerability regardless of migraine type or prior exposure to a different CGRP monoclonal antibody (55–57). RWS have also disclosed greater efficacy than randomized controlled trials (RCT), with rare treatment emergent adverse events, which were mostly mild conditions such as pain, rash or pruritus, flu-like symptoms, and hair loss (56, 58, 59). Additionally, significant reductions in antidepressant and anxiolytic medication use have been observed (60). The PEARL study is currently recruiting to investigate the effectiveness of fremanezumab (61).

Galcanezumab was approved in 2018 (28, 62). It is administered through subcutaneous (SC) injections, with a loading dose of 240 mg in the first month, followed by monthly 120 mg injections. Phase II and Phase III trials EVOLVE-1 and EVOLVE-2 studies showed efficacy and tolerability with similar results as REGAIN study (63–66). Two ongoing studies of galcanezumab are investigating its efficacy in children with chronic and episodic migraines (NCT04616326, NCT03432286). Another recruiting study is the pilot study of galcanezumab in vestibular migraine (NCT04417361). Several RWS are available all showing favorable results, some showing better results than the clinical trials (67–69). In a large RWS nearly 80.0% of the patients reported a decline in the frequency and in intensity of their headaches (70). Another study showed that galcanezumab was effective in chronic migraine regardless of medication overuse and poor responses were correlated with accompanying depression and everyday headache (68).

Eptinezumab has been approved as the first intravenous (iv.) monoclonal antibody (71, 72). Phase II and phase III PROMISE-1 and PROMISE-2 trials showed efficacy and tolerability (73–76). Long-term results of the PREVAIL and DELIVER studies also confirm the safety of eptinezumab (76, 77). There are ongoing studies for the efficacy in MOH (NCT05452239), also for children and adolescents with episodic and chronic migraine (NCT04965675, NCT05164172), as well as one for adult preventive treatment (NCT04921384). However, there is currently no RWS available yet. All monoclonal antibody and gepant drugs are listed in Table 1 and their studies are summarized in Table 2.

**Table 1:** Monoclonal antibodies and gepant drugs.

Drug class	Drug	Brand name	Approval indication	Approval dates			Dosage	C-max/T-max mean	Elimination	Interaction	Adverse effects	Contraindications
				FDA	EMA	Turkiye						
Monoclonal antibodies	Erenumab	Aimovig/Kuzelva	Migraine Prevention	2018	2018	2021	70–140 mg once monthly/SC	15,8–6,1 μg/m(3–11 days)	Saturable binding to target (CGRP-R) in low doses and proteolytic pathway in higher concentrations	N/A	Injection site reactions, pruritus, constipation, muscle spasms, hypertension	Hypersensitivity to the active substance
	Fremanezumab	Ajovy	Migraine Prevention	2018	2019	No availability	225 mg once monthly or 675 mg every 3 months/SC	5–7 days	Catabolic pathways/kidney	N/A	Hypersensitivity reactions, injection site reactions	Hypersensitivity to the active substance
	Galcanezumab	Emgality	Migraine Prevention	2018	2018	2021	240 mg loading dose followed by monthly doses of 120 mg/SC	(28–54 μg/mL) 5 days	Catabolic pathways	N/A	Hypersensitivity reactions, injection site reactions	Hypersensitivity to the active substance
	Eptinezumab	Vjepti	Migraine Prevention	2020	2022	No availability	100–300 mg/IV in approximately 30 min every 3 months.(diluted with %0.9 NaCl)	40.9 (10.9) μg/mL 125 (36.5) μg/mL	Catabolic pathways	N/A	Nasopharyngitis and hypersensitivity, throat irritation, cough, sneezing, dyspnea	Hypersensitivity to the active substance
Gepants	Ubrogepant	Ubrelvy	Acute Migraine Treatment	2019	Not approved	Not approved	50–100 mg during attacks/PO (If needed, a second dose may be administered at least 2 hours after the initial dose)	1.5 (1.0–3.0)	Through CYP3A4	concomitant administration with strong inhibitors of CYP3A4,strong or moderate inducers of CYP3A	nausea, somnolence, nausea	Dose adjustment needed in severe renal and hepatic impairment, contraindicated in end stage renal disease, concomitant use of strong CYP3A4 inhibitors
	Rimegepant	Nurtec ODT/Vydura	Acute/preventive Migraine Treatment	2020	2022	Not approved	75 mg /PO in acute attacks 75 mg every other day in preventive treatment	1–7 (ng/mL)	Through CYP3A4	concomitant administration with strong inhibitors of CYP3A4, strong or moderate inducers of CYP3A, inhibitors of P-gp or BCRP	nausea and abdominal pain/dyspepsia.	Hypersensitivity to the active substance, patients with severe hepatic impairment (see section 4.2); - in patients with end-stage renal disease	
	Zavegepant	Zavzpret	Acute Migraine treatment	2023	Not approved	Not approved	10 mg /intranasal	N/A	Through CYP3A4 and CYP2D6	Avoid using with drugs using OATP1B3 or NTCP transporters, Avoid use in patients with severe hepatic impairment and end stage renal disease	taste disorders, nausea, nasal discomfort, and vomiting	Hypersensitivity reactions
	Atogepant	Qulipta	Acute/preventive Migraine Treatment	2021	Not approved	Not approved	10 mg, 30 mg, or 60 mg/PO	N/A	Through CYP3A4	Dose adjustment with strong CYP3A4 Inhibitor, inducers, Strong and Moderate CYP3A4 Inducers: 30 mg or 60 mg once daily and OATP Inhibitors	Nausea, constipation, and fatigue	None

**Table 2:** Clinical studies of CGRP-targeted treatments.

Drug	Study number	Trial	Time	Indication	Dose	Route of administration	Number of patients	Outcome
Erenumab	NCT01952574	Phase II	12 weeks	Episodic migraine	Monthly SC: 7, 21 mg, 70 m	SC	472	MMDs: 70 mg (−3.4), placebo (−2.3), 7 and 21 mg not significant
	NCT01952574	Extension study	5 years	Episodic migraine	Monthly 140 mg SC	SC	383	MMDs: (−5,3), monthly acute medication use: (−4,4)
	NCT02066415	Phase II	12 weeks	Chronic migraine	Monthly SC: 70 mg, 140 mg	SC	667	MMDs: 70 mg (−6.6) and 140 mg (−6.6), placebo (−4.2)
	NCT02174861	Extension study	52 weeks	Chronic migraine	Monthly SC: 140 mg	SC	609	MMDs: week 40: (−9.3), week 52: (−8.1)
	NCT02456740	Phase III-STRIVE	52 weeks	Episodic migraine	Monthly SC: 70 mg, 140 mg	SC	955	MMDs: 70 mg (−3.2), 140 mg (−3.7), placebo (−1.8)
	NCT02483585	Phase III-ARISE	12 weeks	Episodic migraine	Monthly SC: 70 mg	SC	577	MMDs: 70 mg (−2.9), placebo (−1.8); 50% MMD reduction: 70 mg (40%), placebo (30%)
	NCT03333109	Phase III	12 weeks	Episodic migraine	Monthly SC: 70 mg, 140 mg	SC	900	MMDs: 70 mg (−4,2), 140 mg: (−4.8), placebo (−3,1)
	NCT03096834	Phase III- LIBERTY	12 weeks	Episodic migraine	Monthly SC:140 mg	SC	246	MMDs: 140 mg (−1.8), placebo (−0.2)
	NCT02456740	Phase IV	24 weeks	Head-to-head comparison w topiramate	Erenumab:70 or 140 mg/month/ Topiramat 50-100 mg/day	Erenumab: SC; Topiramate: PO	777	Discontinuation: Erenumab:10.6% vs. Topiramate: 38.9% - ≥50% reduction in MMDs: erenumab:55.4% vs. topiramate:31.2%
Fremanezumab	NCT02025556	Phase II	12 weeks	High frequency episodic migraine	Monthly SC: 225 mg,675 mg	SC	297	MMDs: 225 mg (−6.3), 675 mg (−6.1), placebo (−3.5)
	NCT02021773	Phase II	12 weeks	Episodic migraine	Loading dose 675+ monthly 225 mg, monthly 900 mg, placebo	SC	264	Headache hours: 675/225 mg (−59.8), 900 mg (−67.5), placebo (−37.1)
	NCT02629861	Phase III	12 weeks	Episodic migraine	Single 675 mg, monthly 225 mg	SC	875	MMDs: monthly 225 mg: (−4.0), single 675 mg: (−3.9), placebo: (−2.6)
	NCT02621931	Phase III	12 weeks	Chronic migraine	Single dose 675+ monthly 225 mg, loading dose of 675 mg	SC	1,130	MMDs: monthly 225 mg: (−4.6), single 675 mg: (−4.3), placebo: (−2.5)
	NCT03308968	Phase IIIb- FOCUS	12 weeks	Episodic migraine	Quarterly 675 mg, monthly 225 mg	SC	838	MMDs:quarterly 675 mg: (−3.7), monthly 225 mg: (−4.1), placebo: (−0.6)
Galcanezumab	NCT02163993	Phase II	12 weeks	Episodic migraine/Chronic migraine	Monthly 5, 50, 120, or 300 mg, or placebo	SC	410	MMDs: 120 mg: (−4.3), 300 mg: (−4.3), placebo: (−3.4)
	NCT02614183	Phase III- EVOLVE-1	12 weeks	Episodic migraine	Monthly 240 mg then 120 mg or 240 mg	SC	858	MMDs: 120 mg (−4.7), 240 mg (−4.6), placebo (−2.8)
	NCT02614196	Phase III- EVOLVE-2	6 months	Episodic migraine	Monthly 240 mg then 120 mg or 240 mg	SC	915	MMDs: 120 mg (−4.3), 240 mg (−4.2), placebo (−2.3)	
	NCT02614261	Phase III- REGAIN	12 weeks	Chronic migraine	Monthly SC: 240 mg then 120 mg or 240 mg	SC	1,113	MMDs: 120 mg (−4.8), 240 mg (−4.6), placebo (−2.7)
	NCT03559257	Phase IIIb- CONQUER	12 weeks	Treatment resistant Episodic migraine/Chronic migraine	240 mg loading/ monthly 120 mg, placebo	SC	463	MMDs: 240 mg/120 mg: (−4.1), placebo: (−1.0)
	NCT02614287	Phase III	52 weeks	Episodic migraine/Chronic migraine	Monthly 120 mg, monthly 240 mg	SC	135	MMDs: 120 mg: (−5.6), 240 mg: (−6.5)
Eptinezumab	NCT01772524	Phase II	8 weeks	Episodic migraine	Monthly 1,000 mg	IV	174	Significant reduction in MMDS
	NCT02275117	Phase IIb-	12 weeks	Chronic migraine	Once 10, 30, 100, 300 mg	IV	616	MMDs 75% responder rate: 300 mg (33%), 100 mg (31%), placebo (21%)
	NCT02559895	Phase III- PROMISE-1	12 weeks	Episodic migraine	Once 30, 100, 300 mg	IV	888	MMDs: 300 mg (−4.3), 100 mg (−3.9), placebo (−3.2)
	NCT02974153	Phase III- PROMISE-2	12 weeks	Chronic migraine	Once 30, 100, 300 mg	IV	1,072	MMDs:300 mg (−8.2), 100 mg: (−5.6), placebo (−5.6)
	NCT02559895	Phase III- PREVAIL	48 weeks→extended to 84 weeks	Chronic migraine	300 mg every 12 weeks for 8 doses	IV	128	Safety results of 300 mg
	NCT04418765	Phase IIb- DELIVER	12 weeks	Episodic migraine/Chronic migraine	Once 100, 300 mg	IV	891	MMDs: 300 mg (−5.3), 100 mg (−4.8), placebo (−2.1)
Ubrogepant	NCT02828020	ACHIEVE-1	12 weeks	Acute migraine attacks	Once 50 mg, once 100 mg, placebo	PO	1,672	2 h pain freedom 50 mg: (19.2%), 100 mg: (21.2%), placebo: (11.8%)
	NCT02867709	ACHIEVE-2	12 weeks	Acute migraine attacks	Once 25 mg,50 mg, placebo	PO	1,686	2 h pain freedom 50 mg: (21.8%), 25 mg: (20.7%), placebo: (14.3%)
	NCT02873221	Extension study	52 weeks	Acute migraine attacks	Once 50 mg, once 100 mg, placebo	PO	1,230	Treatment related adverse events 50 mg: (10%), 100 mg: (11%)
Rimegepant	NCT03461757	Phase III	4 weeks	Acute migraine attacks	Once 75 mg, placebo	PO	1,466	2 h pain freedom 75 mg: (21.2%), placebo: (10.9%)
	NCT03732638	Phase II/III	12 weeks	Chronic migraine	Every other day 75 mg, placebo	PO	747	MMDs: 75 mg: (−4.3),placebo: (−3.5)
Zavegepant	NCT03872453	Phase II/III	12 weeks	Acute migraine attacks	5, 10, 20 mg, or placebo	Intranasal	1,673	2 h pain freedom: placebo: (15.5%), 10 mg: (22.5%), 20 mg: (23.1%)
	NCT04571060	Phase III	12 weeks	Acute migraine attacks	10 mg, placebo	Intranasal	1978	2 h pain freedom: placebo: (15%), 10 mg: (24%)
Atogepant	NCT02848326	Phase II/III	12 weeks	Migraine	10 mg,30 mg,60 mg once a day, 30 mg,60 mg twice a day, placebo	PO	1772	MMDs: 10 mg once daily: (−4.0), 30 mg once daily: (−3.8), 60 mg once daily: (−3.6), 30 mg twice daily: (−4.2), 60 mg twice daily: (−4.1), placebo (−2.9)
	NCT03777059	Phase III-ADVANCE	12 weeks	Episodic migraine	Daily 10 mg,30 mg,60 mg, plasebo	PO	2,270	MMDs: 10 mg: −3.7,30 mg: −3.9 60 mg:-4.2,plasebo: −2.5

### 3.2 Gepants

The gepants are a class of small molecule CGRP antagonists that selectively target CGRP receptors. BIBN4096BS was the first gepant investigated in animal studies, followed by a phase I clinical study in 126 patients, which was found to be effective intravenously for acute attacks (78, 79). Olcegepant was the first gepant studied for acute treatment in migraine, showing superiority over placebo in achieving pain freedom within 2 h (80). However, due to severe paresthesia side effects and low bioavailability, it was no longer studied. Telcagepant was the first oral CGRP receptor antagonist found to be effective in RCTs showing promising results as triptans (81, 82). However, in 2014, telcagepant’s use in preventive treatment was halted due to elevations in transaminase levels (83).

Ubrogepant was approved by FDA in 2019 and EMA in 2022 (84, 85). The recommended doses are 50 mg or 100 mg, with an additional dose allowed at least 2 h after the initial dose if needed, and a maximum daily dose of 200 mg. Phase III ACHIEVE-1 study showed significant results with better relief of bothersome symptoms of migraine. Although elevated aminotransferase levels were detected in 6 patients, the liver safety board concluded that this finding was not related to adverse effects of ubrogepant (86). ACHIEVE-2 study also showed similar results (87). The most common adverse effects were nausea and dizziness (88). Safety results showed no treatment-emergent or cardiac adverse events in another *post-hoc* analysis in patients with major cardiovascular risk factors (89). Hepatic and renal insufficiency require dose adjustment, with a maximum daily dose of 100 mg, while end-stage renal failure is a contraindication. An extension trial of 52 weeks comparing the safety and tolerability of 50 mg and 100 mg ubrogepant in 1230 patients, revealed same results as previous studies. Upper respiratory tract infection and nausea were reported. Aminotransferase elevation was reported in 20 patients (90). A study comparing almotriptan and ubrogepant (NCT05214001) is still recruiting, and another study is underway to investigate the efficacy of ubrogepant in adolescents and children (NCT05125302). Also, the combination treatment of ubrogepant with atogepant is still being investigated (NCT05653986). Some combination studies have been completed considering ubrogepant and atogepant, but the data is not available to date (NCT05264129, NCT05653986). One small RWS found similar results to the RCTs concerning the efficacy of ubrogepant, but with higher adverse events (91).

Rimegepant is another CGRP receptor antagonist that received approval from both the FDA in 2020 and the EMA in 2022 for the acute and preventive treatment of migraine (72, 92). The maximum daily dose is 75 mg, and it should be avoided in patients with severe renal or hepatic failure (93). Two phase III trials showed favorable results in safety and tolerability for acute migraine treatment. The most common adverse effects were nausea and urinary tract infections, with no serious adverse effects (94, 95). *Post-hoc* analysis also confirmed these results along with the improved quality of life in patients (96). Long-term safety study showed 75 mg of rimegepant was safe and well-tolerated for the acute treatment of migraine (97). A phase IV study (NCT05211154) is ongoing. Another ongoing phase III study is investigating the long-term safety of rimegepant for the acute treatment of migraine in adolescents and children (NCT04743141). As a phase II/III compared rimegepant 75 mg taken every other day was safe and well-tolerated in the preventive treatment of migraine, it was approved by both the FDA and EMA for the preventive treatment after the studies (92, 98, 99). More investigations of rimegepant are continuing, including both the efficacy and tolerability of rimegepant for the acute and preventive treatment of migraine in adults (NCT05509400, NCT05518123) as well as RWS (NCT05709106). In a study comparing galcanezumab and rimegepant; galcanezumab was not found to be superior to rimegepant; however, both interventions demonstrated efficacy as preventive treatments (100). Another ongoing phase III RCT is evaluating the safety and tolerability of preventive migraine treatment in adolescents and children (NCT05156398). To date, no RWS has been published on the efficacy and safety of rimegepant.

Zavegepant, formerly known as vazegepant, is the first intranasally administered CGRP receptor antagonist that has been approved by the FDA in 2023 in acute migraine treatment (101). A completed phase II/III of zavegepant 10 mg and 20 mg were found to be more effective than placebo in both areas. The most common adverse effects were dysgeusia, nausea, and nasal discomfort No hepatotoxicity was detected (102). Another phase III trial showed favorable results in pain freedom and freedom from the most bothersome symptom (103). Long-term safety and tolerability results of zavegepant were shown by a phase II/III open-label trial (NCT04408794) (104). Another phase II/III study is currently recruiting, investigating the efficacy and safety of zavegepant in the preventive treatment of migraine (NCT04804033).

Atogepant is an FDA-approved oral CGRP receptor antagonist for the preventive treatment of episodic migraine, available in doses of 10 mg, 30 mg, or 60 mg (105). Mild-to-moderate renal and hepatic impairment does not require dose adjustment, while severe hepatic disease is a contraindication. In cases of severe renal impairment or concurrent use of CYP3A4 inhibitors, the maximum daily dose is limited to 10 mg. The first phase II/III trial showed favorable results considering mean decrease in MMDs (106). Nausea, constipation, and fatigue were the most common adverse events, and no liver toxicity was observed. The phase III ADVANCE study showed statistically significant reductions of MMDs (107). A phase III open RCT showed constipation in atogepant group (108). The extended results of the ADVANCE trial over 40 weeks demonstrated that 60 mg of atogepant daily was safe and well-tolerated (109). An observational diary study evaluating the real-world effectiveness of the acute treatment of migraine with ubrogepant when used in combination with atogepant for prevention is planned to finish in April 2024 (NCT05653986). Another study assessing the change in disease activity when ubrogepant and atogepant tablets are combined to treat migraine in adult participants is ongoing and planned to finish in April 2024 (COURAGE II; NCT05653986). There is currently no RWS available for atogepant.

## 4 Current guidelines

Migraine treatment is guided by various international societies, including those from the International Headache Society (HIS), American Headache Society (AHS), European Headache Federation (EHF), and National Institute of Healthcare and Excellence (NICE). The development of new CGRP-based agents has led to significant changes in migraine treatment perspectives, resulting in the issuance of consensus statements and new treatment guidance by these societies (21, 110–112). In addition, the Health Services General Directorate of Turkiye Ministry of Health published guidelines for migraine treatment in 2020 and is planned to be rearranged (113).

The treatment guidelines for acute migraine vary among different organizations (Table 3). EHF recommendation is stepped care approach, while AHS recommendation is mostly a stratified approach (21, 114). British Association for the Study of Headache (BASH) and Turkiye recommend either a stratified therapy or a stepped care approach depending on the severity of the attack (111, 113). Even in the absence of nausea and vomiting, antiemetics are recommended by NICE and BASH. While EHF and NICE advice against oral ergot alkaloids, the AHS and Turkiye guidelines include ergot derivatives in the acute migraine treatment as patient-based decision in refractory cases. NICE suggests beginning triptan treatment with the least expensive available option, and all guidelines provide an alternative triptan if the primary one is ineffective. NICE and AHS also included neuromodulatory devices in selected cases which showed good tolerability and safety (112, 115, 116). The BASH, NICE, and Turkiye guidelines were established before the development of ditans and gepants; therefore, not yet included in the guidelines. There are ongoing preparations to incorporate gepants and ditans into these guidelines.

**Table 3:** Acute treatment recommendations in guidelines.

Treatments	EHF	AHS	NICE	BASH	THMR
Only NSAIDs, Simple analgesics	1				
Only Triptans	2				
Combination of Triptans +NSAIDs or Paracetamol	2/4				
Gepants + Ditans	3/4	4	7		
Opioids					4
Antiemetics	6	6	5	5	5
Neuromodulatory devices		4	4		

NICE, National Institute for Health and Care Excellence; AHS, American Headache Society; BASH, Brain Attack Study in Hypertension; THMR, Turkiye Health Ministry Migraine Report; EHF, European Headache Federation; Green, Recommended (patient based); Red, Not Recommended; Gray, Not mentioned, ^1^First line Treatment, ^2^Second Line Treatment, ^3^Third line treatment, ^4^In case of inability or unresponsiveness to other treatments, ^5^in case of nausea, ^6^Even nausea is absent, ^7^Rimegepant only.

The preventive treatment guidelines for migraine differ across various medical societies, much like the acute treatment guidelines (Table 4) (21, 111, 112, 117). Up to date guidelines have not yet included eptinezumab and rimegepant for the preventive treatment, despite the fact that rimegepant is approved for migraine prevention by both FDA and EMA. NICE suggests the use of monoclonal antibodies in patients with four or more migraine days in a month who have an inadequate response to three preventive drug treatments. If the frequency does not reduce by 50% in episodic migraine or 30% in chronic migraine after 12 weeks, the treatment is recommended to be stopped. Transcranial magnetic stimulation is recommended in NICE for selected cases (118). AHS also suggests monoclonal agents in patients with four or more migraine days in a month. It is offered in cases of insufficient treatment with traditional agents for 8 weeks or more, if Migraine Disability Assessment (MIDAS) score is more than 11, headache impact test (HIT)-6 > 50 in episodic and chronic migraine, or chronic migraine patients who are unable to tolerate or show inadequate response to a minimum of two quarterly injections (6 months) of onabotulinumtoxinA. Although frovatriptan is not approved as a preventive therapy, it has been included as a preventive choice of medication in AHS guidelines, probably due to its long effect duration. EHF updated the recommendation on the use of monoclonal antibodies targeting the CGRP pathway for migraine prevention as they are effective and safe also in the long-term. The EHF expert panel provides the most detailed guide for CGRP monoclonal antibodies use in preventive treatment for migraine. Investigations and preparations for the new guideline are in progress in Turkiye.

**Table 4:** Preventive treatment recommendations in guidelines.

Drugs	EHF	AHS	NICE	BASH	THMR
Beta blockers without intrinsic sympathomimetic activity	1	5			
Topiramate	1	5			
Candesartan	1	5			
Valproic acid	2	5			
Flunarizine	2				
Amitriptyline	2	6			
Monoclonal antibodies	1	5	4		
Botulinum toxin	3	6	4		
Gepants as preventive treatment		7			
Riboflavin					
Frovatriptan		7			
Memantin		6			
Lisinopril		6			
Venlafaksin		6			
Neuromodulatory devices, biobehavioural therapy and acupuncture					
Diltiazem					
Verapamil					
Gabapentin					
Pregabaline					
Zonisamide					
Siproheptadin					

NICE, National Institute for Health and Care Excellence; AHS, American Headache Society; BASH, Brain Attack Study in Hypertension; THMR, Turkiye Health Ministry Migraine Report; EHF, European Headache Federation; Green, Recommended (patient based); Red, Not Recommended; Gray, Not mentioned; ^1^First line Treatment, ^2^Second Line Treatment, ^3^Third line treatment, ^4^In case of inability or unresponsiveness to other treatments ^5^Established Efficacy, ^6^Probably Effective, ^7^Identified as dual use agents with meaningful benefits as both acute and preventive treatments; no guidance on use yet.

## 5 Obstacles and barriers to achieve optimal migraine treatment and suggestions to overcome them

### 5.1 Accurate diagnosis and appropriate treatment

Population-based studies as CaMEO and OVERCOME studies have demonstrated that the majority of migraine patients lack an accurate diagnosis and appropriate treatment (119, 120). In a study conducted in Turkiye, it was reported that 22.8% of patients with a definitive diagnosis of migraine had previously been diagnosed with tension-type headache, and 37.1% of patients diagnosed with tension-type headache had previously been diagnosed with sinusitis (121). In another study conducted in our country, 40.9% of migraine patients stated that they had previously been diagnosed with tension-type headache. Possible explanations for this difference include the symptoms not meeting definitive migraine diagnostic criteria in previous physician evaluations, the variable nature of migraine, or the alternation between migraine and tension-type headache (122). In Turkiye, headache treatment is offered through governmental hospitals, university hospitals free of charge for individuals covered by social security, as well as private hospitals. Unlike some other countries, patients in Turkiye, have the ability to directly consult a neurologist for headache complaints, although visit durations are typically very brief within the social security system. The neurology consultation periods are shortened due to intense patients load, the initial diagnosis and management of numerous migraine patients is believed to be incomplete. The MIRA-Neurology study group stated that headache complaint caused at least 1/3 of all neurological outpatient visits in Turkiye and 2/3 of all patients admitted to neurology clinics had headache (123). This suggests that awareness of migraine in our society may have grown over time, leading to a shift in patient preference toward consulting neurologists. Such a trend is highly beneficial as it promotes early initiation of appropriate treatment for migraine patients, potentially lowering rates of chronicity. Examining the global data from the “My Migraine Voice” health survey, it was found that the initial healthcare provider sought by patients was typically a general practitioner (53%) (124). This contrasts with the trends observed in Türkiye, possibly due to the emphasis on encouraging patients to seek care from primary health services, a distinction from healthcare policies in other nations. In terms of workforce loss, the rates of patients reporting that migraine affects their professional lives were notably higher in Türkiye compared to global data (125). While the burden of migraine in Türkiye is similar to other countries, the remarkable high number of emergency room visits compared to other countries imposes a significant financial and operational strain on the “free” healthcare system provided by the social security system. To alleviate this burden, it is necessary to create special guidelines for primary care physicians, thus the diagnosis and treatment of migraine patients will not be initiated only by neurologists. Additionally, these guidelines may outline algorithms for determining when patients should be referred to specialized centers. The existing guidelines and algorithms for neurologists in Turkiye, encompassing all the latest treatments, should be revised and updated. Regular and standardized effective trainings are crucial to keep neurologists well-informed and updated about the diagnosis and treatment of migraine. Furthermore, there is a necessity to enhance the scientific content available to doctors on social media platforms, ensuring it is both increased and kept current. It is strongly advisable for hospitals to establish headache outpatient clinics with specialized physicians if they are not already in place. This helps prevent inaccurate or incomplete diagnoses and ensures optimal patient care.

### 5.2 Safer acute treatments

Studies from Turkiye showed only 43.1% of the patients were using medications under the supervision of a physician and only 2.9% of them were using migraine specific medication during acute attacks (121). Migraine-specific treatments are subject to prescription limits in some countries similar to Turkiye leading to inadequate access and treatment with triptans. This results in patients transitioning to other over-the-counter (OTC) analgesics, leading to OTC related MOH (125, 126). Although ergot alkaloids are not used in Europe and United States, they are still being prescribed for moderate–severe attacks in selected cases. Despite this, patients tend to use “cheap” drugs regardless of the attack severity in Turkiye. Furthermore, commercial availability of many triptans is restricted, greatly reducing the effectiveness of acute attack treatments. On the contrary, unlike many countries the available triptans can be purchased without prescription which may lead to MOH eventually. Despite the licensing of erenumab, and galcanezumab in Turkiye, obtaining these drugs remains challenging for patients due to lack of insurance coverage, it creates a significant barrier to access to medicines for patients in need. Efforts should be made to enhance accessibility to all acute treatment agents in our country. Effective treatment of not only the acute attack of migraine but also the accompanying symptoms is important for the quality of life of patients. Patients, hindered by photophobia are unable to drive and may find themselves in distressing situations due to nausea. To avert such scenarios, there is a need for treatments that can swiftly and effectively halt the migraine attack. The drugs to be used during acute attack treatment should not have the potential for side effects that could affect the daily life of patients.

### 5.3 Improved preventive therapies

Research indicates that only 10% of individuals receive preventive treatment, despite the fact that preventive medication could benefit over 40% of episodic migraine sufferers (127). Similarly, it was reported that only 4.9% of the patients received prophylactic treatment in Turkiye, although the monthly number of attacks was four and above in more than half of the patients with migraine (121). The timely initiation of preventive treatment in eligible patients will also serve to mitigate MOH resulting from excessive drug usage. Preventive treatments that are rapidly effective, devoid of potential side effects, and do not necessitate a wash-out period are in demand.

### 5.4 Understanding the underlying mechanisms

CGRP antagonists, the sole migraine-specific compounds at present, seem to be currently positioned at the bottom among all available treatments. The reason for this is primarily attributed to their high cost and the absence of reimbursement policies, which significantly limit access for many individuals. Furthermore, the long-term effects of most molecules are unknown, and speculation suggests that long-term CGRP suppression with the injectable molecules may have negative effects on cardiovascular health, bone density, and immune function (128). Erenumab and galcanezumab have been approved for use in Turkiye, patients continue to have difficulty accessing these drugs because of the lack of full coverage of these drugs by social security agency and private insurance companies. These obstacles suggest a need for more streamlined and affordable migraine treatment options in Turkiye. Advancements in technology allow for a clearer understanding of the mechanisms behind existing treatments, and ongoing studies on migraine pathophysiology have the potential to contribute significantly to the development of more targeted and specific treatments for migraines.

### 5.5 Personalized treatment approaches

Tailoring treatment plans to individual patient characteristics remains a challenge. Every treatment modality should be evaluated closely, particularly in chronic migraine patients. The discontinuation of migraine prophylaxis is also an important consideration in this context. It is crucial to individually assess the response to treatment, any encountered adverse effects, and the overall efficacy of the preventive therapy in managing migraine symptoms, taking into account the specific needs of the patient (129).

#### 5.5.1 Genetic predispositions

Many genetic and environmental factors are involved in the etiology of migraine. It has been stated that hereditary factors contribute to the etiology of migraine in the range of 34 to 57%. However, the genetic background is not well defined due to its inherent heterogeneity. Studies suggest that the *5-HTR2C rs3813929 and TNF-a-308G/A* polymorphism can be a genetic risk factor for migraine in the Turkish population (130, 131). Pain perception and response mechanisms also differ among individuals and in HEAD-MENAA study, lifetime pain duration of the patients admitted to neurology clinics was significantly longer in the Middle East and Turkiye than in the other regions (132). Through advancements in precision medicine, healthcare providers aim to craft treatment plans that optimize efficacy while minimizing side effects for each patient. This approach extends beyond focusing solely on symptom relief, encompassing the broader aspects of a patient’s life, including their emotional well-being and daily functioning and aims to improve quality of life.

#### 5.5.2 Addressing comorbidities

Migraine often coexists with other conditions and diseases, such as mood disorders, sleep disturbances and cardiovascular events (133). In addition to medical interventions for sleep disorders and stress, patients can be guided toward cognitive-behavioral treatments. Integrating comprehensive care that addresses these comorbidities is a key unmet need. Managing and treating the underlying comorbid conditions play a significant role in minimizing migraine attacks and enhancing the efficacy of treatments.

#### 5.5.3 Multidisciplinary and holistic approaches

Involves addressing not only the acute symptoms but also considering the broader aspects of a patient’s physical, emotional, and social well-being. Holistic care includes lifestyle modifications, stress management, dietary considerations, and other non-pharmacological interventions alongside traditional medical treatments. As migraine is a condition characterized by multi-aspect and complex pathophysiology, its treatment should not be confined solely to neurologists. Instead, a multidisciplinary assessment involving other physicians is essential, considering the underlying psychosocial conditions. Support from non-medical professionals, such as dietitians, psychologists, and life coaches, is valuable to provide comprehensive care (134). In addition to the patient’s adherence to migraine treatment, patient’s treatment response is likely to improve with lifestyle modifications, including addressing sleep issues, managing screen exposure, practicing stress management, engaging in regular exercise, and adjusting diet.

### 5.6 Patient education and access

Social determinants, geographic and geo-economic disparities, economic burden, cultural misconceptions, limited resources and overburdened healthcare services, and stigma are the main factors in difficulty to access to migraine treatment by taking into account a local and global scale (135, 136). Economically, disparities in income and healthcare coverage significantly impact individuals’ ability to access headache treatment. Despite Turkiye providing free access to public healthcare systems and diagnostic examinations, prolonged waiting periods may ensue due to the large influx of patients. Disparities in headache diagnosis, treatment, and management become particularly apparent, especially in rural regions lacking sufficient healthcare resources. Furthermore, the absence of insurance coverage for certain medications including CGRP monoclonal antibodies and specialized treatments presents a barrier for low-income patients seeking adequate treatment. Hence, patient education and awareness is also an essential component of migraine management. In a study, adding patient education to routine migraine medical treatment resulted in a reduction in mean headache days per month and a greater reduction in functionally incapacitating headache days per month, less analgesic overuse, increased adherence therapy, and made fewer headache-related calls to the clinic (137). Patients, in particular, ought to trust their physicians and adhere to the prescribed treatments consistently. In Turkiye, patients frequently rely on medications suggested by their social circle, and some opt to directly visit the pharmacy instead of consulting a doctor. They purchase medications and use them inconsistently. Empowering patients with education enable them to make informed decisions about their health and engage more effectively in their treatment plans. The establishment of migraine-specific patient associations and support groups is essential. Health authorities and associations should disseminate accurate information through brochures, social media, and TV announcements to enhance awareness and support.

**Figure 3:**
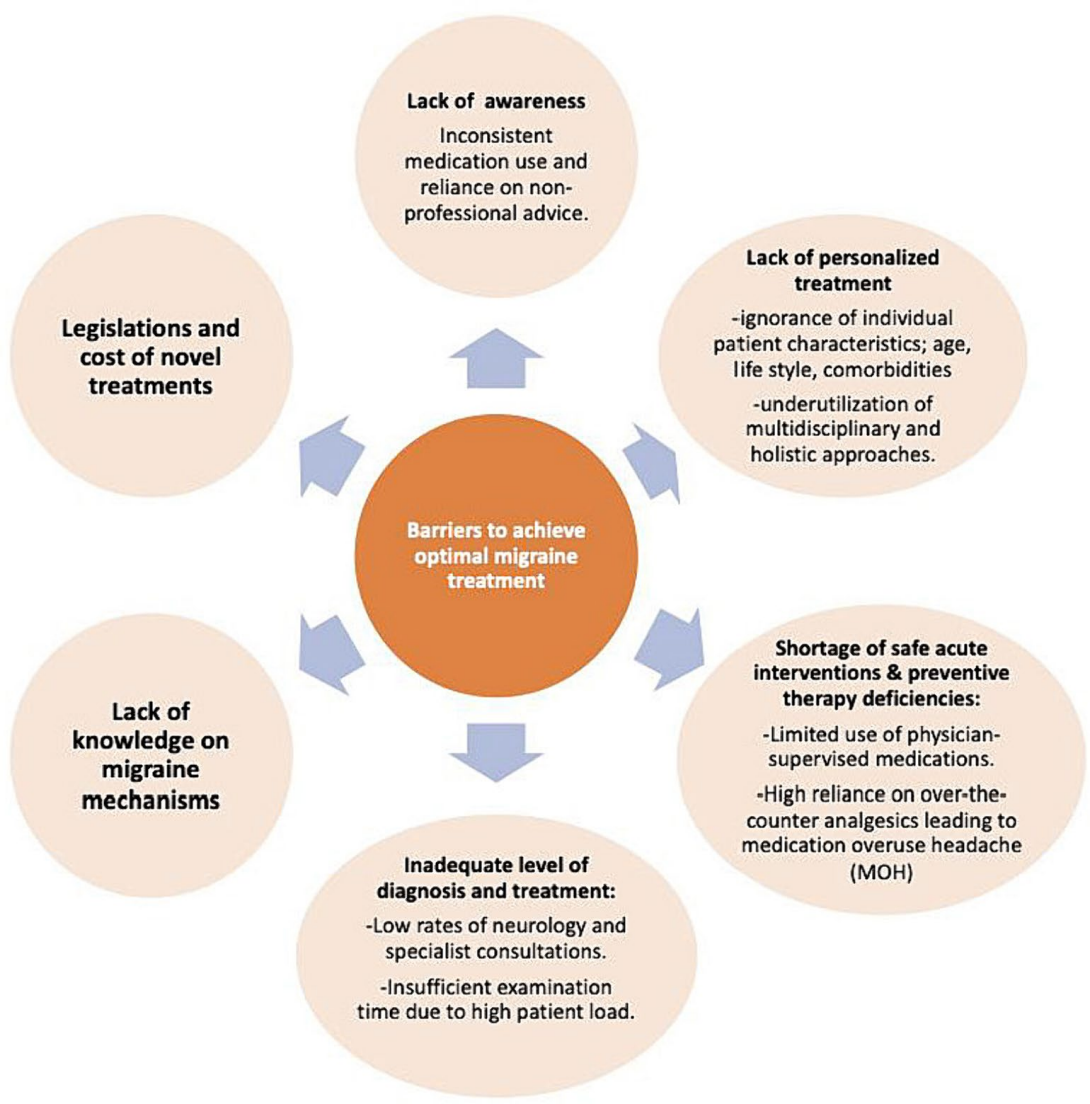
Barriers to achieve optimal migraine treatment.

**Figure 4:**
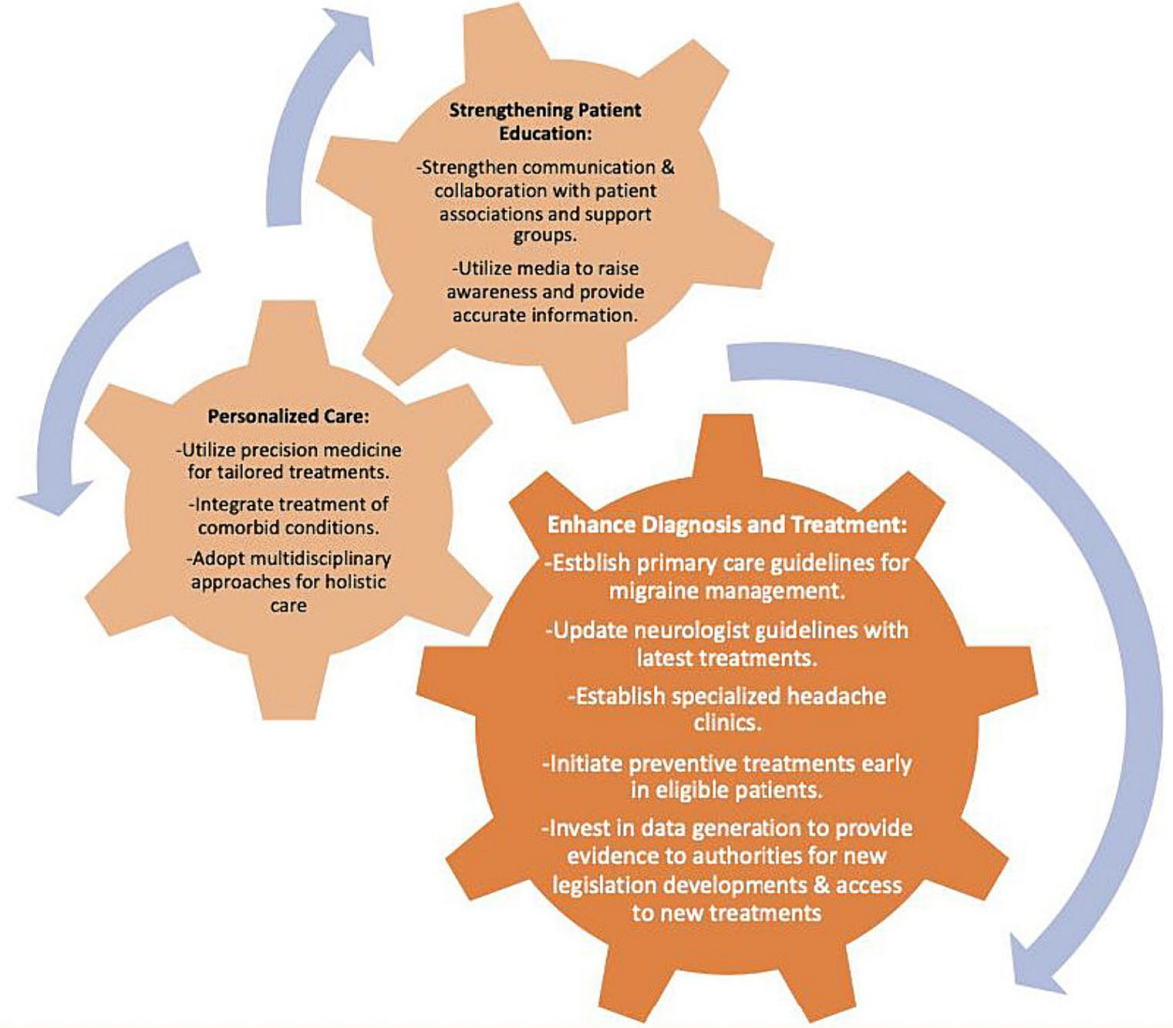
Suggestions to improve treatment management in migraine.

Barriers to achieve optimal migraine treatment and suggestions to improve treatment management in migraine, respectively, presented in Figures 3, 4.

In light of recent advancements in migraine research and treatment modalities, it is imperative to engage in a comprehensive discussion regarding the challenges of managing migraine in the contemporary healthcare landscape. Personal viewpoints play a pivotal role in shaping the discourse surrounding migraine management, as they provide insights into individual experiences, preferences, and treatment outcomes. By fostering a more engaging dialog, healthcare professionals can collaboratively explore the multifaceted challenges encountered in migraine care, including medication adherence, treatment efficacy, and the impact of comorbidities on overall management strategies. Furthermore, addressing patient-specific concerns and incorporating patient perspectives into treatment decisions are essential components of a patient-centered approach to migraine care. Through open and transparent communication, coupled with a thorough understanding of the evolving therapeutic landscape, healthcare providers can navigate the complexities of migraine management more effectively, ultimately improving patient outcomes and quality of life.

## 6 Conclusion

Obstacles in migraine treatment include poor specification in migraine therapies, lack of tailored treatment plans, accurate diagnosis and appropriate treatment eventually leading to MOH, inadequate time in evaluation due to high rate of outpatient clinic visit numbers, limited access to specialists, unavailability of some of the new treatment options for effective acute and preventive treatment. Researchers have sought innovative strategies for migraine specified therapies such as CGRP-targeting drugs. However, high costs and limited long-term data on these drugs present challenges to widespread adoption. Other strategies to overcome the aforementioned obstacles can be listed as establishing guidelines and algorithms for primary care physicians, establishing diseases specified outpatient clinics in available centers, sharing the latest scientific content with doctors and help them keep updated with the new medications, efforts in enhancing accessibility of new treatment options in all countries, education of patients about treatment options, changes of life style and holistic approaches. Also creating patient groups and informative messages through mass media by the health authorities can be useful.

## Author contributions

AÖ: Writing – review & editing. BB: Writing – review & editing. ŞB: Writing – review & editing. ME: Writing – review & editing. AA: Writing – review & editing. SG: Writing – review & editing. NK: Writing – review & editing.
